# The diagnostic and prognostic implications of *PRKRA* expression in HBV-related hepatocellular carcinoma

**DOI:** 10.1186/s13027-022-00430-6

**Published:** 2022-06-21

**Authors:** Yi-Min Hu, Ruoxi Ran, Chaoqi Yang, Song-Mei Liu

**Affiliations:** 1 Department of Clinical Laboratory, Hunan Provincial People’s Hospital, The First Affiliated Hospital of Hunan Normal University, Changsha, 410005 China; 2 Department of Clinical Laboratory, Center for Gene Diagnosis, and Program of Clinical Laboratory Medicine, Zhongnan Hospital of Wuhan University, 169 Donghu Road, Wuhan, 430071 Hubei China

**Keywords:** Hepatitis B virus-related hepatocellular carcinoma (HBV-related HCC), *PRKRA*, *EIF2AK2*, Inflammatory cytokines, Biomarker

## Abstract

**Background:**

Hepatitis B virus (HBV)-related hepatocellular carcinoma (HCC) accounts for more than half of total HCC patients in developing countries. Currently, HBV-related HCC diagnosis and prognosis still lack specific biomarkers. Here, we investigated if *PRKRA* expression in peripheral blood could be a potential biomarker for the diagnosis/prognosis of HBV-related HCC.

**Methods:**

The expression of *PRKRA* in HBV-related HCC was firstly analyzed using TCGA and GEO databases. The results were confirmed in a validation cohort including 152 blood samples from 77 healthy controls and 75 HCC patients, 60 of which were infected with HBV. The potential diagnostic and prognostic values of *PRKRA* were also evaluated by the area under the receiver operator characteristic curve (AUROC) and Kaplan–Meier method, respectively.

**Results:**

*PRKRA* was significantly upregulated in HCC patients, especially in those with HBV infections. In addition, the combination of *PRKRA* expression in peripheral blood with serum AFP and CEA levels displayed a better diagnostic performance (AUROC = 0.908, 95% CI 0.844–0.972; *p* < 0.001). Notably, when serum AFP is less than 200 ng/mL, *PRKRA* expression demonstrated better diagnostic capability. Furthermore, *PRKRA* expression levels were associated with expression of *EIF2AK2* and inflammatory cytokine genes.

**Conclusions:**

Triple combination testing of blood* PRKRA* expression, serum AFP and CEA levels could be a noninvasive strategy for diagnosis; and the elevation of *PRKRA* expression could predicate poor prognosis for HBV-related HCC.

**Supplementary Information:**

The online version contains supplementary material available at 10.1186/s13027-022-00430-6.

## Introduction

Hepatocellular carcinoma (HCC) is the sixth most common cancer globally and the second leading cause of cancer-related mortality. Hepatitis B virus (HBV) is one of the most significant risk factors for HCC, especially in HBV epidemic regions [1]. Patients with HBV-associated cirrhosis have a 31-fold increase risk of HCC and 44-fold increase mortality compared to non-cirrhosis patients [2]. In addition, several studies have identified HBV-related factors as crucial predictors of HCC development in patients with chronic hepatitis B, such as serum positive HBV E antigen, high viral load, and viral genotype [2]. Despite an effective vaccine, about 257 million people were infected with HBV [3, 4]. Thus, HBV-related HCC still presents a worldwide threat to public health.

HCC is often diagnosed at advanced stages. The earlier diagnosis was beneficial for the prognosis of HCC since it allowed an early treatment, including surgical resection and a noninvasive ablation. X-ray, biopsy, histopathology, CT/MRI, and serum AFP level are commonly used for HCC diagnosis in clinical practice. Patients with chronic hepatitis were suggested to accept abdominal ultrasound examination together with detection of serum AFP [2, 5]. Nonetheless, current tests with limitations in sensitivity and specificity could result in false positive or inconclusive results. Although advances in cancer care and treatment have improved patient survival, the clinical outcomes of HCC remain dismal, with a less than 50% five-year survival rate after diagnosis [6]. Therefore, it is urgent to identify specific molecules that regulate carcinogenesis and find out potential biomarkers for HCC diagnosis, especially HBV-related HCC.

A double-stranded RNA binding protein (PACT) is encoded by the *PRKRA* gene. Like argonaute-1 (AGO1), argonaute-2 (AGO2) and TARBP2, PACT belongs to the RNA-induced silencing complex (RISC) [7]. PACT and TARBP2 interact with mammalian DICER to form a complex, which can bind to the ∼70 nt long pre-miRNA “hairpin”, and cleave the loop to produce a double-stranded RNA [8]. Increasing evidence has revealed that these major miRNA pathway components, including *PRKRA,* were dysregulated in cancers such as epithelial skin cancer and non-small cell lung carcinoma [9, 10]. In addition, PACT was a known cellular protein activator of PKR kinase (also known as *EIF2AK2*) in a dsRNA‐independent manner in response to cellular stress [11, 12]. PKR could regulate protein translation and trigger the integrative stress response via an eIF2α kinase dependent manner to enhance translation of transcription factors such as ATF4 [13]. Moreover, PACT could enhance the function of retinoic acid-inducible gene I (RIG-I) and induce the expression of type I interferons (IFNs). Both melanoma differentiation-associated gene 5 (MDA5) and laboratory of genetics and physiology 2 (LGP2) were necessary to activate interferon signaling in response to host cell infection by various viruses, including ebola virus (EBOV), influenza A virus, Middle East respiratory syndrome coronavirus, human T-cell leukemia virus 1, mouse hepatitis virus, measles virus, and herpes simplex virus 1 [13, 14].

Recently, *PRKRA* has been reported to improve oxaliplatin sensitivity in mucinous ovarian cancer cells [15]. The SNP rs2059691 was also associated with increased mRNA expression of *PRKRA* and worse survival of colorectal cancer patients [16]. However, few studies focused on the link between *PRKRA* and HCC, especially in HBV-related HCC.

In this study, we firstly found upregulation of *PRKRA* expression in HBV-related HCC and suggested that the expression of *PRKRA* in peripheral blood could be a potential biomarker for the diagnosis and prognosis of HBV-related HCC.

## Materials and methods

### Participants

The *PRKRA* expression in tumor tissues and clinical information were obtained from The Cancer Genome Atlas (TCGA) dataset (https://portal.gdc.cancer.gov/) and the GEO database (http://www.ncbi.nih.gov/geo). The TCGA cohort contained 371 tumor tissues (HBV-positive group, n = 145; HBV-negative group, n = 226) and 50 tumor-adjacent tissues. The GEO dataset (GSE19665) included 5 pairs of tumor and tumor-adjacent tissues. The validation cohort included 152 blood samples from 75 HCC patients (60 cases of HCC with HBV infection; 64 males and 11 females, mean age 56.5 ± 10.2), and 77 healthy controls (35 males and 42 females, mean age 54.2 ± 9.7) (Additional file 1: Table S1). HCC patients were confirmed by pathology. HCC patients with HBV infection were defined as HBV-related HCC according to serology testing results. The detailed information on liver function and serum tumor biomarkers are described in Additional file 1: Table S1.

### Blood RNA extraction and qPCR

Total RNA was isolated from peripheral white blood cells by Trizol reagent (Invitrogen, Carlsbad, USA). RNA was quantified using spectrophotometry (NanoDrop 2000, NanoDrop Technologies, Inc., Wilmington, DE), and the RNA integrity was verified by agarose gel electrophoresis.

The cDNA was synthesized with a reverse-transcription kit with DNAase (Toyobo Co. Ltd., Osaka, Japan). The mRNA expression of *PRKRA* and *EIF2AK2* were determined in triplicates using iTaq™ Universal Supermixes (Bio-Rad, Hercules, CA) with normalization to *GAPDH* and *β-ACTIN*. Primer sequences were obtained from Primer Bank (http://pga.mgh.harvard.edu/primerbank) shown in Additional file 1: Table S2.

### Statistical analysis

The SPSS software (version 21.0, IBM Inc., Chicago, IL) was used to analyze all data. Comparisons between groups were performed with the Mann–Whitney U test. The prognosis was evaluated by the Kaplan–Meier method and log-rank test. And univariate and multivariate Cox regression models were employed to assess independent prognostic factors. The pairwise deletion method was used to deal with missing data. All the *p* values were two-sided, and the statistical significance level was at α = 0.05. (*p < 0.05, **p < 0.01, ***p < 0.001.)

## Results

### *PRKRA* expression is upregulated in HBV-related HCC

**Fig. 1:**
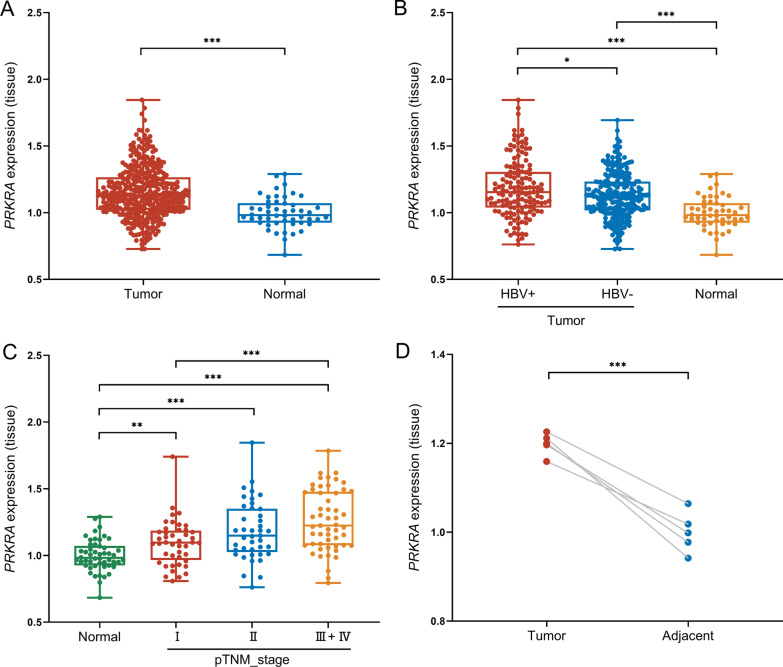
*PRKRA* is upregulated in HBV-related HCC. **A**, **B** *PRKRA* mRNA expression in HCC tumor tissues (HBV-positive group, n = 145; HBV-negative group, n = 226) and tumor-adjacent tissues (n = 50). Data were derived from TCGA datasets. **C** The *PRKRA* mRNA expression levels in controls and at different TNM stages of HBV-related HCC patients (control, n = 50; stage I, n = 44; stage II, n = 41; stage III + IV, n = 53). **D** *PRKRA* mRNA expression in matched tumor and tumor-adjacent tissues from 5 patients. Data were derived from GSE19665 dataset

As shown in Fig. 1A, B, the expression of *PRKRA* was significantly upregulated in HCC tumor tissues, especially in the HBV-positive group (*p* < 0.001). Moreover, the expression of *PRKRA* was higher in the advanced TNM-stage group than the early TNM-stage group (*p* < 0.001) (Fig. 1C). We then compared the mRNA expression levels of *PRKRA* in tumor tissues and tumor-adjacent tissues from GSE19665 dataset (n = 5), and these results confirmed the increase expression of *PRKRA* in HCC (*p* < 0.001) (Fig. 1D).

### Higher *PRKRA* expression levels predict a poor prognosis in HBV-related HCC

**Fig. 2:**
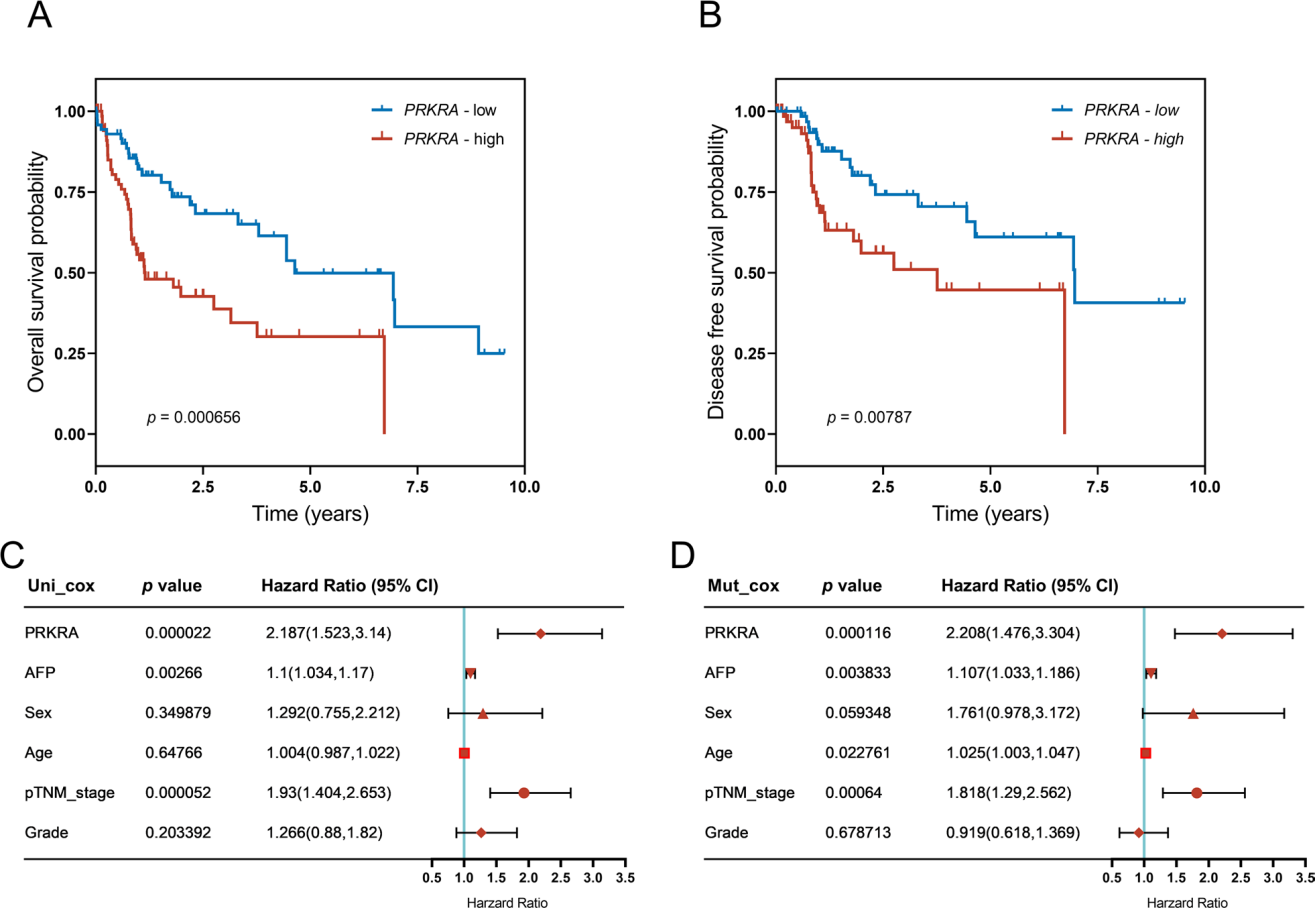
High *PRKRA* expression levels predict poor prognosis of HBV-related HCC patients. **A**, **B** Kaplan–Meier analysis of overall survival (**A**) and disease-free survival (**B**) of HBV-related HCC patients based on *PRKRA* expression (n = 145). The median of *PRKRA* expression levels was used as the cutoff value for grouping **C**, **D** Univariate analysis (**C**) and multivariate COX regression models (**D**) including 6 parameters (*PRKRA* expression, serum AFP level, sex, age, TNM-stage and pathologic grade) were employed to explore risk factors for HBV-related HCC. Symbols indicate Hazard ratio, and bars indicate 95% CIs in forest plots. Uni: Univariate; Mut: multivariate; CI: confidence interval

Next, we analyzed the association between *PRKRA* expression and prognosis of HBV-related HCC patients. The patients were divided into *PRKRA*-low and *PRKRA*-high groups according to the expression levels of *PRKRA* in tumors. The Kaplan–Meier analysis indicated that patients in the *PRKRA*-high group had a poor overall survival (Fig. 2A; log-rank *p* value < 0.001) and disease-free survival (Fig. 2B; log-rank *p* value < 0.001) than patients in the *PRKRA*-low group. Then univariate analysis and multivariate Cox regression models including six parameters (*PRKRA* expression*,* AFP levels, sex, age, TNM-stage and pathologic grade) were performed. The results showed that *PRKRA* expression was correlated with patients' clinical outcomes and was an independent risk factor for HBV-related HCC patients (Hazard ratio = 2.208, 95% CI 1.476–3.304, *p* < 0.001). Collectively, these results indicate that *PRKRA* could be a prognosis predictor for HBV-related HCC.

### The diagnostic performance of *PRKRA* in peripheral blood as a biomarker for HBV-related HCC

**Fig. 3:**
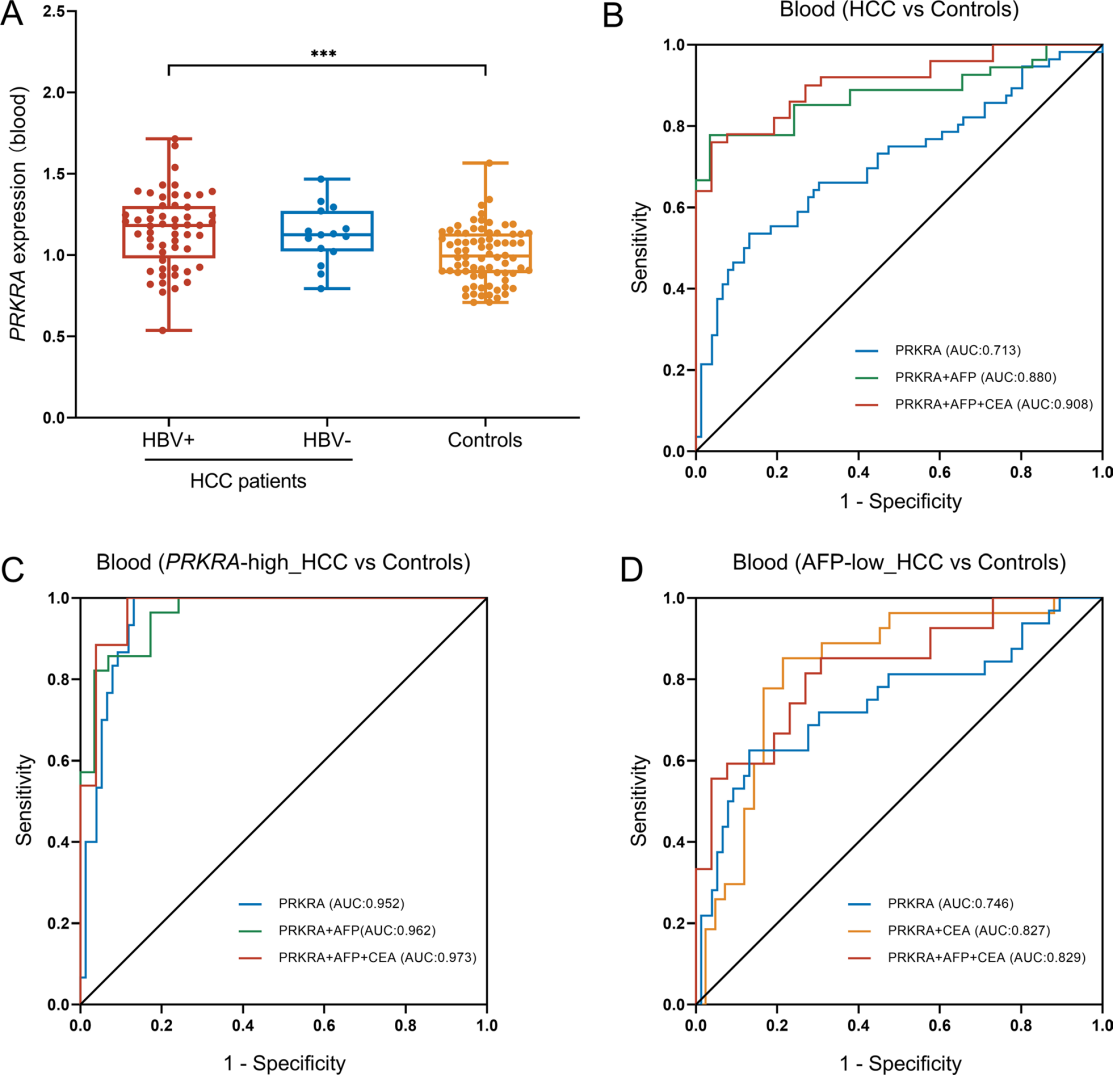
*PRKRA* expression in peripheral blood has the potential diagnostic capability for HBV-related HCC. **A** *PRKRA* mRNA expression in the validation cohort including 152 blood samples from 77 healthy controls and 75 HCC patients, 60 of which were infected with HBV. **B** AUROCs for *PRKRA*, *PRKRA* + AFP, *PRKRA* + AFP + CEA in HBV-related patients and healthy controls. **C** According to the cut-off value (2.341), patients were divided into *PRKRA*-high and *PRKRA*-low groups. AUROCs for *PRKRA*, *PRKRA* + AFP, *PRKRA* + AFP + CEA in *PRKRA*-high group. **D** In HBV-related patients with AFP ≤ 200 ng/mL and healthy controls, AUROCs for *PRKRA*, *PRKRA* + CEA, *PRKRA* + AFP + CEA

The increased expression of *PRKRA* in HCC, especially in HBV-related HCC was further confirmed in 152 blood samples from HCC patients and healthy controls (Fig. 3A and Additional file 1: Fig. S1). Then the AUROC was plotted to test whether the *PRKRA* expression in peripheral blood had diagnostic capacity for HBV-related HCC. As shown in Fig. 3B, *PRKRA* expression in peripheral blood could distinguish HCC patients from healthy controls with AUROC of 0.713 (95% CI 0.621–0.805; *p* < 0.001). *PRKRA* expression combining with serum AFP and CEA showed a much higher AUROC (*PRKRA* + AFP: 0.880, 95% CI 0.806–0.952; *p* < 0.001; *PRKRA* + AFP + CEA: 0.908, 95% CI 0.844–0.972; *p* < 0.001). Notably, the diagnostic sensitivity of *PRKRA* expression was 54% (cutoff value = 2.341), while *PRKRA* expression, serum AFP and CEA served as a combined diagnostic indicator for HBV-related HCC could increase sensitivity to 76%. Besides, *PRKRA* expression values ≥ 2.341 showed a better diagnostic value (*PRKRA*: AUROC = 0.952, 95% CI 0.914–0.990; *p* < 0.001; *PRKRA* + AFP: AUROC = 0.962, 95% CI 0.920–1.00; *p* < 0.001; *PRKRA* + AFP + CEA: AUROC = 0.973, 95% CI 0.934–1.00; *p* < 0.001). These findings revealed that *PRKRA* expression, serum AFP and CEA could act as a combined diagnostic indicator for HBV-related HCC.

Next, we paid attention to the diagnostic potential of *PRKRA* in patients with AFP ≤ 200 ng/mL. *PRKRA* expression levels in peripheral blood (AUROC = 0.746, 95% CI 0.633–0.858; *p* < 0.001) indicated a better diagnostic capability than serum AFP (AUROC = 0.626, 95% CI 0.477–0.775; *p* = 0.095) and CEA (AUROC = 0.682, 95% CI 0.558–0.806; *p* = 0.010) (Fig. 3C). In addition, a combination of *PRKRA* expression, AFP and CEA could improve the diagnostic capability when serum AFP was at a low level in HBV-related HCC patients. These data suggested that the *PRKRA* expression in peripheral blood provided the potential diagnostic capability for HBV-related HCC patients.

### *PRKRA* expression levels are associated with *EIF2AK2* and inflammatory cytokine genes

To explore the possible reasons for increased *PRKRA* expression and poor prognosis in HBV-related HCC, we compared the mRNA expression of *EIF2AK2* in tumor tissues and blood samples from the HBV-related HCC patients. Similar to the expression pattern of *PRKRA, EIF2AK2* was also upregulated both in tumor tissues and peripheral blood samples (Additional file 1: Fig. S2). Increased *EIF2AK2* expression levels were also associated with the poor prognosis of HBV-related HCC patients (Additional file 1: Fig. S3).

**Fig. 4:**
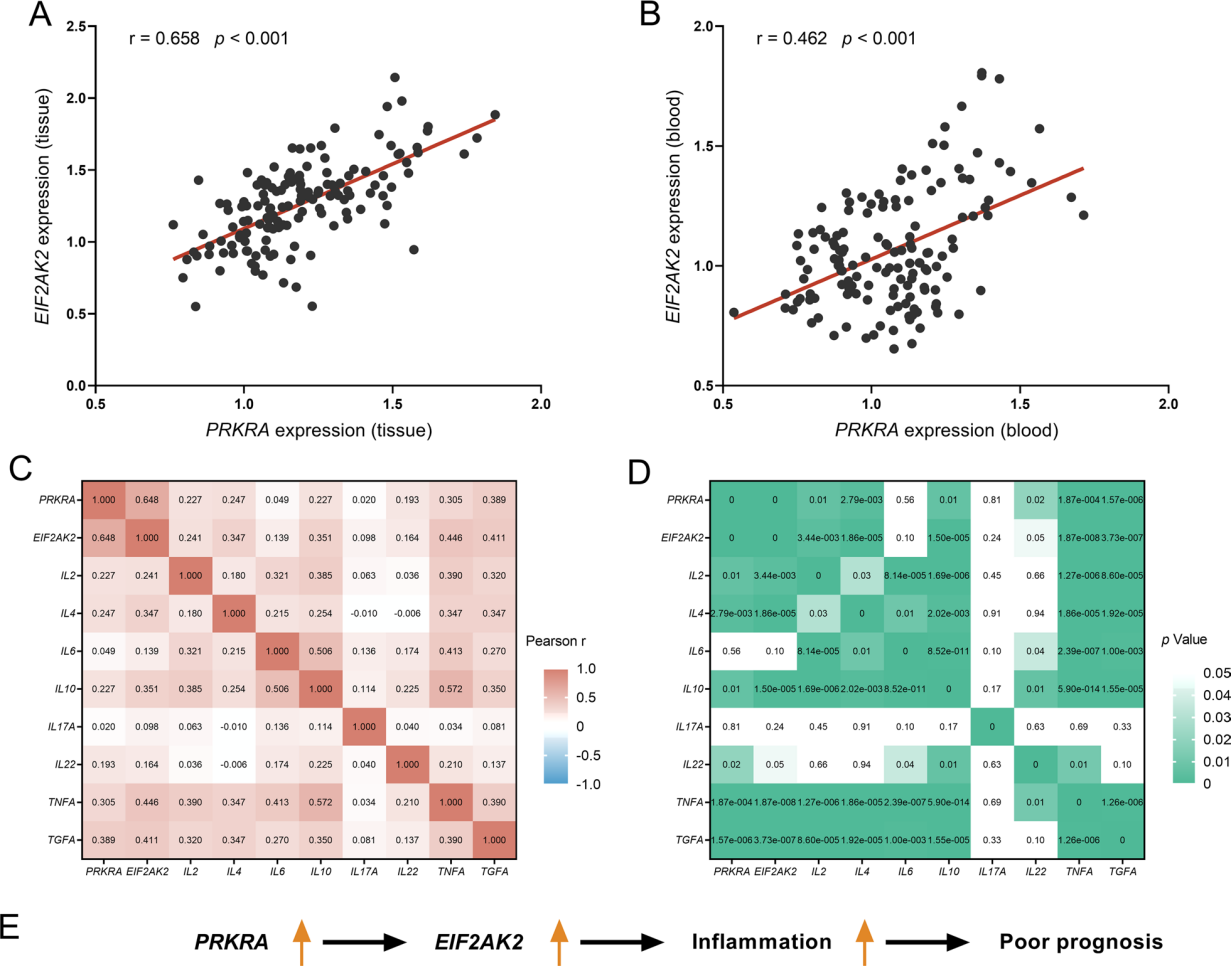
*PRKRA* expression levels are associated with *EIF2AK2* and inflammatory cytokines. **A**, **B** The positive correlation between *PRKRA* expression levels and *EIF2AK2* expression levels in tissues (**A**) and peripheral blood samples (**B**). **C**, **D** Heatmaps indicating the associations across *PRKRA*, *EIF2AK2* and inflammatory cytokines genes. Pearson r and *p* values shown in **C**, **D**, respectively. Data were derived from TCGA database. **E** A schematic illustration of *PRKRA* upregulation was associated with *EIF2AK2* and inflammation, which explain the possible reasons for poor prognosis in HBV-related HCC

Pearson correlation analysis was then used to investigate the link between *PRKRA*, *EIF2AK2* and inflammatory cytokine genes (i.e., *IL-2*, *IL-4*, *IL-6*, *IL-10*, *IL-17*, *IL-22*, *TNF-α*, *TGF-α*) through a TCGA database including 145 HBV-positive patients. A significant positive correlation between *PRKRA* expression and *EIF2AK2* expression was observed in tissues (r = 0.658, *p* < 0.001; Fig. 4A) and peripheral blood samples (r = 0.462, *p* < 0.001; Fig. 4B). Moreover, *PRKRA* and *EIF2AK2* were both positively correlated to the expression of inflammatory cytokine genes, including *IL-2* (r = 0.227, *p* = 0.01; r = 0.241, *p* < 0.001), *IL-4* (r = 0.247, *p* < 0.001; r = 0.347, *p* < 0.001), *IL-10* (r = 0.227, *p* = 0.01; r = 0.351, *p* < 0.001), *TNF-α* (r = 0.305, *p* < 0.001; r = 0.446, *p* < 0.001) and *TGF-α* (r = 0.389, *p* < 0.001; r = 0.441, *p* < 0.001) (Fig. 4 C, D). These results indicated that *PRKRA* / *EIF2AK2* could lead to poor clinical outcomes through activating inflammatory response (Fig. 4F).

## Discussion

HBV-related HCC has been a global health problem that accounts for more than half of total HCC patients in developing countries [17, 18]. In the present study, we found an increase of *PRKRA* expression in both tumor tissues and peripheral blood samples in HBV-related HCC. The *PRKRA* expression in peripheral blood combined with serum AFP and CEA displayed a potential diagnostic efficacy with an AUROC of 0.908. To our knowledge, this result is the first time to show the potential diagnostic and prognostic values of *PRKRA* in HBV-related HCC.

Serum AFP has been the most common biomarker for HCC screening in the last decades, with a sensitivity of 41–65% and a specificity of 80–94% (cutoff value = 20 ng/mL). However, AFP was not secreted by all hepatoma cells, and it might also increase in some patients with cirrhosis or hepatitis [19]. Evidence has shown that nearly half of HCC patients were AFP-negative, especially at an early stage and in small HCCs [19]. Therefore, we focused on the diagnostic efficacy of *PRKRA* for patients with serum AFP ≤ 200 ng/mL. Interestingly, *PRKRA* showed a better diagnostic capability than serum AFP.

Furthermore, we also found *PRKRA* expression was associated with *EIF2AK2* and inflammatory cytokine genes, leading to poor prognosis in HBV-related HCC. *PRKRA* is a known activator of PKR kinase, which plays an essential oncogenic role in HCC. A recent study found that LINC00665, a long intergenic noncoding RNA that physically interacted with PKR, was involved in the NF-κB signaling activation and promoted hepatic cancer progression [20]. PKR activation in stimulated hepatic stellate cells could also promote the development of HCC [21]. In HCC with hepatitis C virus infection, PKR upregulated c-Fos and c-Jun activities to accelerate tumor development [22]. The PKR inhibitor C16 was discovered to block HCC tumor cell growth and angiogenesis in vitro and in vivo through a decrease of growth factors [23]. Mouse xenograft models also confirmed the tumorigenic role of PKR in HepG2 cells by activating STAT3 [24]. Furthermore, *PRKRA* could bind to the PKR kinase domain and produce interferon and cytokines in virally infected cells [25]. Inflammatory responses to stress can be inhibited by targeting the interaction between PKR and *PRKRA* [26]. Notably, inflammation was a recognized marker of cancers that substantially contributed to the progression of malignancies, including HBV-related HCC [27]. Consequently, it is reasonable to surmise that the elevated inflammation activated by the increased interaction between *PRKRA* and *EIF2AK2* leads to poor prognosis of HBV-related HCC.

In conclusion, the current study elucidates the elevated expression of *PRKRA* in HBV-related HCC. The increased *PRKRA* expression was associated with *EIF2AK2* and inflammation, which could explain the possible reasons for poor prognosis in HBV-related HCC. Triple combination testing of blood PRKRA expression and serum AFP and CEA levels could be a noninvasive strategy for the diagnosis and prognosis of HBV-related HCC.

## Supplementary Information

**Additional file 1**.** Supplementary Table S1**. Clinical characteristics of 152 blood samples from HCC patients and healthy controls.** Supplementary Table S2**. Primers for qRT-PCR used in this study.** Supplementary Figure S1**. PRKRA is up-regulated in HCC compared with non-HCC and healthy controls. **Supplementary Figure S2**. EIF2AK2 is up-regulated in HBV-related HCC. **Supplementary Figure S3**. Higher EIF2AK2 expression levels are associated with a poor prognosis of HBV-related HCC.

## Data Availability

All data are reported in the manuscript.

## Abbreviations

HCC: Hepatocellular carcinoma
HBV: Hepatitis B virus
AGO1: Argonaute-1
AGO2: Argonaute-2
RISC: RNA-induced silencing complex
dsRNA: Double-stranded RNA
RIG-I: Retinoic acid-inducible gene I
IFNs: Type I interferons
MDA5: Melanoma differentiation-associated gene 5
LGP2: Laboratory of genetics and physiology 2
TCGA: The Cancer Genome Atlas
