# Supplementary material for: The diagnostic and prognostic implications of PRKRA expression in HBV-related hepatocellular carcinoma
Source: Infect Agent Cancer. 2022 Jun 21;17:34. doi: 10.1186/s13027-022-00430-6 (PMC9211784; doi:10.1186/s13027-022-00430-6)

**Supplementary Materials**

**Clinical Laboratory Tests**

Liver functions (alanine aminotransferase [ALT], aspartate aminotransferase [AST]) were determined with the AU5800 Chemistry Analyzer (Beckman, California, USA). Serum tumor biomarkers (α-fetoprotein [AFP], carcinoembryonic antigen [CEA]) were assayed by the i4000SR Immunology Analyzer (Abbott Lab, Chicago, USA).

**Supplementary Tables**

**Supplementary Table S1**：Clinical characteristics of 152 blood samples from HCC patients and healthy controls.

**Supplementary Table S2**：Primers for qRT-PCR used in this study.

**Supplementary Figure legend**

**Supplementary Figure S1**：***PRKRA* is up-regulated in HCC compared with non-HCC and healthy controls.** PRKRA expression in blood were tested by qRT-PCR and compared among HCC (n= 75), non-HCC (n= 75) and control (n= 77) groups.

**Supplementary Figure S2**：***EIF2AK2* is up-regulated in HBV-related HCC.** (A) *EIF2AK2* mRNA expression in a panel of HCC tissue specimens (HBV+ group, n = 145; HBV- group, n=226) and adjacent tissues (n = 50). Data were derived from TCGA datasets. (B) *EIF2AK2* mRNA expression in matched tumor and adjacent tumor tissues from 5 patients. (C) The *EIF2AK2* mRNA expression levels in controls and different TNM stages of HBV-related patients (control, n = 50; stage I, n = 44; stage II, n = 41; stage III + IV, n = 53). (D) *EIF2AK2* mRNA expression in validation cohort including 152 blood samples from 77 healthy controls and 75 HCC patients, 60 of which were infected with HBV.

**Supplementary Figure S3**：**Higher *EIF2AK2* expression levels are associated with a poor prognosis of HBV-related HCC.** (A-B) Kaplan-Meier analysis of overall survival (A) and disease-free survival (B) of HBV-related HCC patients based on *EIF2AK2* expression (n = 145). The cut off values for grouping were determined by the median of *EIF2AK2* expression levels.

Table S1 Clinical characteristics of 152 HCC patients and healthy controls.

| **Parameter** | **HCC** | | **Controls (n=77)** | **p value** |
| --- | --- | --- | --- | --- |
|  | **HBV+ (n=60)** | **HBV- (n=15)** |  |  |
| Gender |  |  |  |  |
| Male, % | 55(91.7%) | 9(60.0%) | 35(45.5%) | <0.001^*^ |
| Female, % | 5(8.3%) | 6(40.0%) | 42(54.5%) |  |
| Age, years | 56.0(50.0, 63.0) | 60.0(50.0, 65.0) | 50.5(47.0, 61.8) | 0.050 |
| **Liver function** |  |  |  |  |
| ALT, U/L | 36.50(24.00,55.75) | 28.50(16.00,58.00) | 17.50(14.00,21.75) | <0.001^*^ |
| AST, U/L | 45.50(32.25,91.50) | 37.00(25.75,79.00) | 20.50(18.00,25.00) | <0.001^*^ |
| **Tumor biomarkers** |  |  |  |  |
| AFP, ng/mL | 141.71(3.07,2720.17) | 3.46(2.67,303.05) | 2.87(2.30,4.20) | <0.001^*^ |
| CEA, ng/mL | 2.24(1.59,2.92) | 2.43(1.75,5.24) | 1.60(1.09,2.70) | 0.005^*^ |

Abbreviation: HCC, hepatocellular carcinoma; HBV, hepatitis B virus; ALT, alanine aminotransferase; AST, aspartate aminotransferase; AFP, a-fetoprotein; CEA, carcinoembryonic antigen.

Data are expressed as median (interquartile range).

Difference among groups was tested using the χ^2^ test for proportions and Kruskal-Wallis test for continuous variables.

^*^*p* < 0.05.

Supplemental Table S2 Primers for qRT-PCR

| **Gene** | **Primers (5ʹ -3ʹ)** |
| --- | --- |
| *PRKRA* | Forward: CTGCTTAGTGAAATTGCCAAGGA  Reverse: GAGCCATGACAGACTGTGATG |
| *EIF2AK2* | Forward: TGGAAAGCGAACAAGGAGTAAG  Reverse: CCAAAGCGTAGAGGTCCACTT |
| *GAPDH* | Forward: TCTATAAATTGAGCCCGCAGC  Reverse: CCAATACGACCAAATCCGTTG |
| *ACTB* | Forward: CCAGCTCCTCCCTGGAGAAG  Reverse: ACAGGACTCCATGCCCAGG |

Figure S1


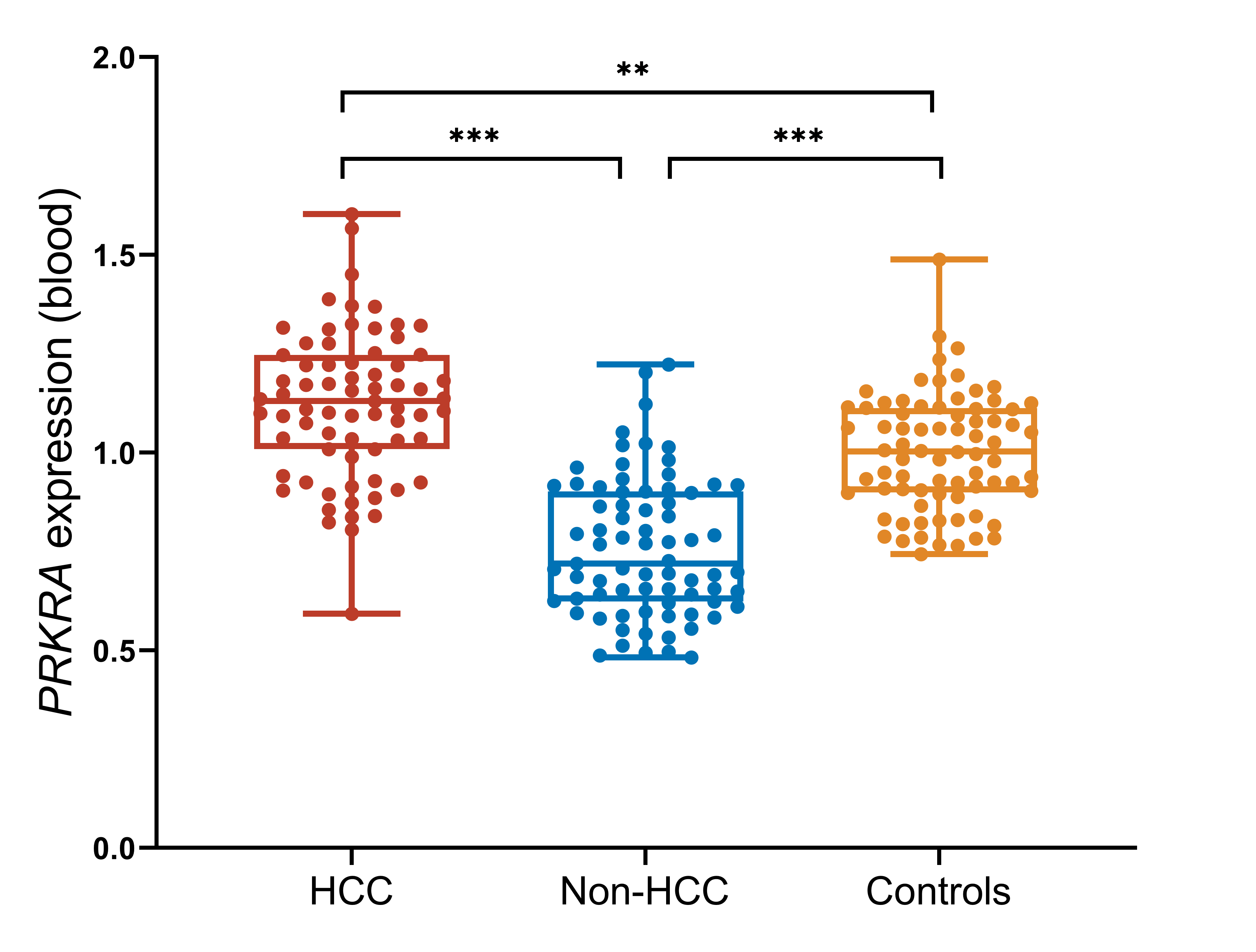


Figure S2


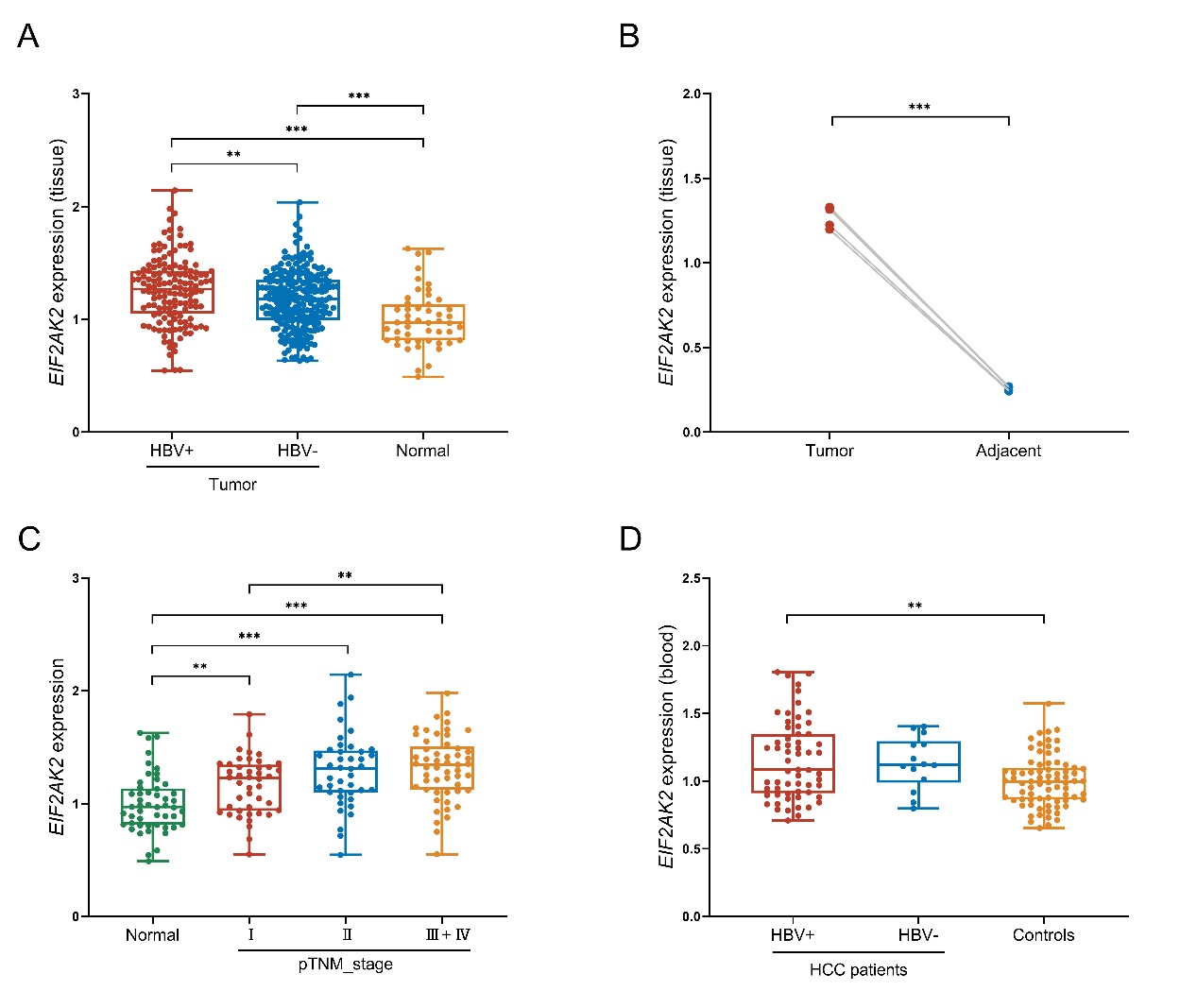


Figure S3


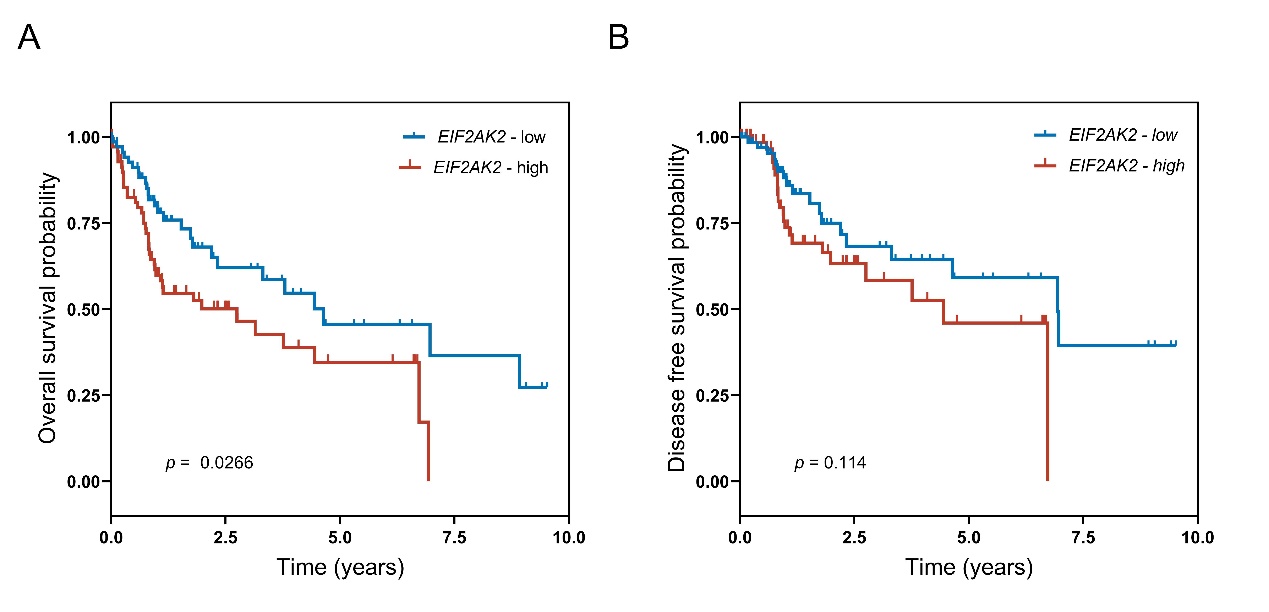

Supplement: Supplementary file 1 — Additional file 1. Supplementary Table S1. Clinical characteristics of 152 blood samples from HCC patients and healthy controls. Supplementary Table S2. Primers for qRT-PCR used in this study. Supplementary Figure S1. PRKRA is up-regulated in HCC compared with non-HCC and healthy controls. Supplementary Figure S2. EIF2AK2 is up-regulated in HBV-related HCC. Supplementary Figure S3. Higher EIF2AK2 expression levels are associated with a poor prognosis of HBV-related HCC. [file 13027_2022_430_MOESM1_ESM.docx]
